# Socioeconomic Influences on the Outcomes of Dialysis-Requiring Acute Kidney Injury in Brazil

**DOI:** 10.1016/j.ekir.2023.06.003

**Published:** 2023-06-14

**Authors:** Conrado Lysandro Rodrigues Gomes, Thais Lyra Cleto-Yamane, Frederico Ruzani, José Hermógenes Rocco Suassuna

**Affiliations:** 1 Clinical and Academic Unit of Nephrology, Hospital Universitário Pedro Ernesto, Faculty of Medical Sciences, Universidade do Estado do Rio de Janeiro, Rio de Janeiro, Brazil; 2 Kidney Assistance Ltd., Rio de Janeiro, Brazil

**Keywords:** acute kidney injury, dialysis, epidemiology, socioeconomic

## Abstract

**Introduction:**

Although research suggests that socioeconomic deprivation is linked to a higher incidence of acute kidney injury (AKI) and worse outcomes in high-income countries, there is limited knowledge about these epidemiologic factors in developing countries. In addition, the impact of medical institution administration (private versus public) on AKI outcomes remains to be determined.

**Methods:**

We studied 15,186 pediatric and adult patients with dialysis-requiring AKI (AKI-D) admitted to private and public hospitals in Rio de Janeiro, Brazil. According to Brazil's demographic census, socioeconomic indicators were derived from patient zip codes. Propensity score matching analysis and a mixed-effect Cox regression were used to assess the impact of socioeconomic indicators and hospital governance on patient survival.

**Results:**

Crude mortality rates were higher in private hospitals than in public hospitals (71.8% vs. 59.5%, *P* < 0.001) and were associated with significant differences in age (75 years, interquartile range [IQR]: 61–83 vs. 53 years, IQR: 31–66), baseline renal function (prevalence of chronic kidney disease [CKD]: 33.2% vs. 23%, *P* < 0.001), comorbidities (Charlson score: 2.03 ± 0.87 vs. 1.72 ± 0.75, *P* < 0.001), and severity of presentation (mechanical ventilation: 76.5% vs. 58% and vasopressors: 72.8% vs. 50.5%, *P* < 0.001). After adjustments and propensity score matching, we found no effect of different hospital administrations or socioeconomic factors on mortality. Baseline characteristics and the severity of presentation primarily influenced AKI-D prognosis.

**Conclusions:**

Despite significant racial and socioeconomic differences in hospital governance, these indicators had no independent influence on mortality. Future epidemiologic studies should investigate these relevant assumptions to allow healthcare systems to manage this severe syndrome promptly.

## Graphical abstract

**Figure:**
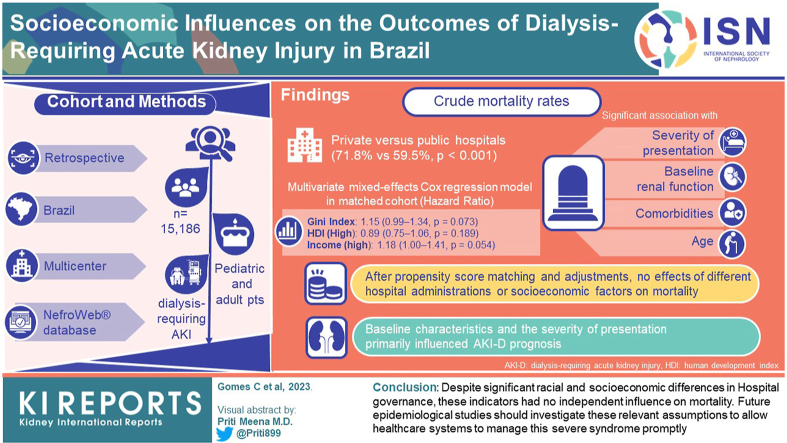

AKI remains associated with poor short-term and long-term outcomes and rising treatment costs.^1^ Therefore, obtaining high-quality data on the epidemiology of AKI worldwide, not only in its many clinical aspects but also in its socioeconomic determinants, is critical to assisting physicians and healthcare policymakers in consistently and sustainably managing this critical syndrome.^2^^,^^3^

For many disease conditions, the influence of socioeconomic differences on health outcomes has been documented. For example, studies from different countries with different healthcare delivery models have consistently found an association between CKD and socioeconomic deprivation.^4^ However, the impact of these factors in the AKI setting has received far less attention, despite the fact that low socioeconomic status has been linked to an increased risk of AKI and poor patient outcomes.^5^, ^6^, ^7^, ^8^, ^9^, ^10^ Information is even scarcer in developing countries, where health information systems may be deficient or inaccurate.^3^ Moreover, AKI epidemiology may differ within the same country because of differences in sanitation, disease distribution, and access to quality healthcare.^11^^,^^12^

One factor contributing to disease outcomes is the disparity in quality of care provided by the public and private sectors when they coexist in 2-tiered healthcare systems. Unfortunately, it is difficult to distinguish which factors are related to the type of provider and which are influenced by demographic, environmental, and educational or economic factors.^13^^,^^14^

This study investigates the epidemiologic features and associated outcomes of patients with AKI-D in a large metropolitan area of Brazil, focusing on the performance of healthcare institutions that are either market-oriented private or government-funded sector. We also analyzed the impact of other socioeconomic factors (education, income, longevity, human development, and inequality) on AKI outcomes.

## Methods

### Study Protocol

We performed a retrospective analysis of the NefroWeb database, an 11 year (2002–2012) prospective, multicenter study on clinical characteristics and outcomes of AKI-D in the metropolitan area of Rio de Janeiro, Brazil.^15^ The study included 170 private and public hospitals and medical institutions. Personally sponsored or company-sponsored private health plans or insurance (market oriented) were the primary source of funding in private governance hospitals. In public governance hospitals, the primary source of financing was the National Healthcare System (Sistema Unico de Saúde), and it was provided free of charge at the point of consumption. After nephrology consultation and AKI-D diagnosis, data were entered into the clinical module of NefroWeb, a customized Microsoft SQL Server database, by the attending nephrologist at the time of renal replacement therapy (RRT) initiation.^15^ Because the study was noninterventional, the institutional research ethics board approved the study protocol and waived the need for informed consent. This paper follows the STROBE guidelines for cohort studies (Supplementary File S1, Appendix)

### Study Population

**Figure 1:**
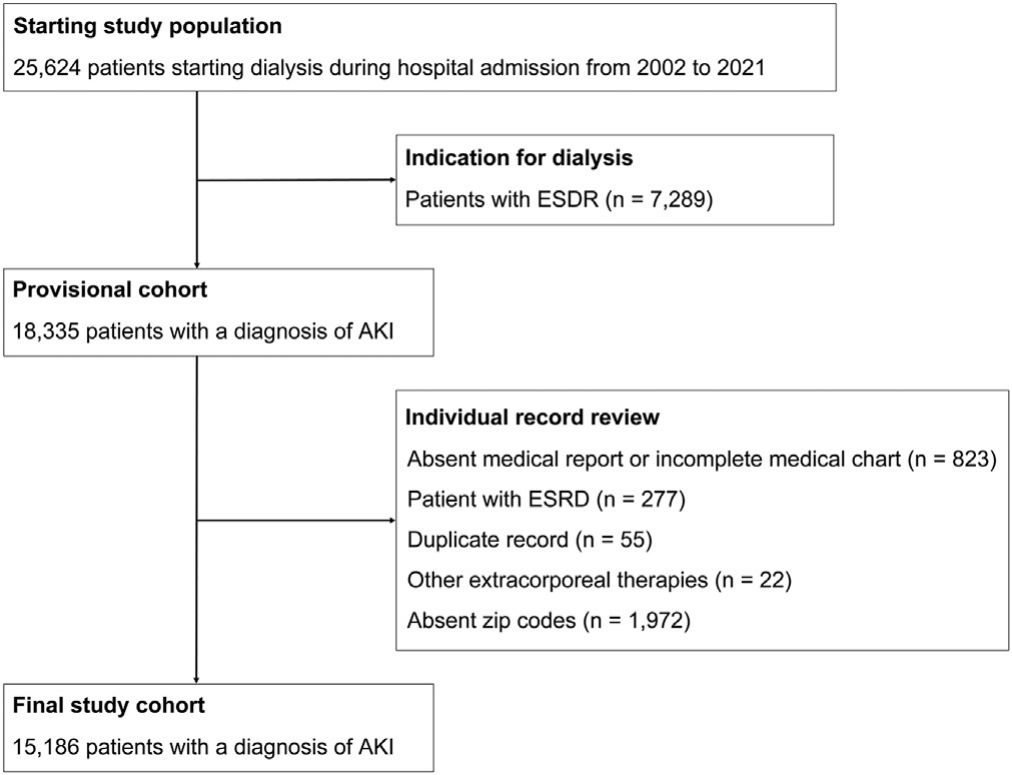
Study flowchart.

All adult or pediatric patients, admitted to one of the 170 hospitals and medical facilities, and required dialysis during hospitalization were eligible for inclusion in this cohort. Out of the 25,624 patients who were dialyzed, we excluded 7289 patients diagnosed with end-stage kidney disease (either on previous chronic outpatient RRT or patients with CKD who were commencing dialysis treatment at the time of hospital admission). After chart review, we excluded 3149 patients with incomplete reports, missing values, or missing zip codes (which were required to compute socioeconomic data), leaving the final cohort of 15,186 patients (Figure 1).

### Data Collection

The study protocol was previously described in detail.^15^ Briefly, we collected patient demographic data (age, sex, and race) and clinical variables (primary clinical or surgical diagnosis, renal diagnosis, and comorbidities [the Charlson comorbidity score was derived from the database's extensive list of comorbidities and clinical diagnoses available in the database], precipitant cause of AKI-D, and severity of presentation and organ failures), AKI-D phenotype (e.g., *de novo* AKI vs. acute-on-chronic kidney disease [ACKD] and hospital-acquired vs. community-acquired AKI), dialysis indication, and modality at the time of dialysis initiation. By conducting detailed medical histories and laboratory studies at enrollment, we identified and excluded patients with CKD who were beginning dialysis at the time of hospital admission. This allowed us to differentiate them from patients with *de novo* AKI or underlying nondialytic ACKD. Categorization of the patients with ACKD was done on the day of nephrology evaluation based on available information, such as previous medical records, admission serum creatinine, past laboratory results (when available), medical history, relatives' or caregivers' interviews, kidney sonography, and clinical judgment. As a result, the patients were categorized as having either *de novo* AKI or ACKD. Dialysis modalities included continuous automated peritoneal dialysis and hemodialysis with standard machinery in the following methods: conventional intermittent hemodialysis, prolonged intermittent RRT (PIRRT), or PIRRT in continuous mode (meaning continuous hemodialysis provided with standard dialysis equipment and PIRRT set-up parameters).^16^ After nephrology RRT indication, data were entered into the clinical module of NefroWeb by the attending nephrologists (for more information on the NefroWeb database, data collection process, definitions used [e.g., ACKD, sepsis, cardiorenal syndrome, and liver failure definitions] and dialysis modalities; see Supplementary File S1, sections 1 and 2). At hospital discharge, the primary outcomes were death, complete or partial recovery, and dialysis dependence. Complete recovery was defined as serum creatinine returning to the same level or a lower level than the enrollment value, whereas partial recovery was defined as serum creatinine being lower than the peak but higher than the enrollment value. In addition, hospitals were classified based on their governance (private vs. public), bed capacity (small: <50 beds; medium: 50–150 beds; and large: >150 beds), and specialty (general, pediatric, cardiology, emergency/trauma, obstetrics, oncology, and infectious diseases). Finally, each record was reviewed by a pediatric nephrologist (TLCY) or adult nephrologist (CLRG or JHRS) for implausible values, event ascertainment, adjudication, and data cleaning.

### Socioeconomic Indicators

We recovered the following variables based on the geographic region of individual patient residence derived from postal zip codes: global municipal human development index (HDIM), further divided into 3 categories (education, income, and longevity), Gini index for socioeconomic disparities, and mean monthly per capita income. Data were presented as continuous variables and categorized in terciles or dichotomized based on mean values in the state of Rio de Janeiro. The United Nations Development Program in Brazil, the Institute for Applied Economic Research, and the João Pinheiro Foundation adapted the country's overall human development index to calculate these indicators for municipalities and states, which are available on the website http://atlasbrasil.org.br (data from the 2010 census). The HDIM is interpreted in the same way as the overall human development index, but its components aim to adapt the global human development index methodology to Brazilian municipalities at municipal and state levels. It has a scale of 0 to 1 and allows for the comparison of Brazilian cities over time.

### Statistical Analysis

Analyses were performed using the R statistical environment, version 4.2.2, with a significance level set at *P* < 0.05. Categorical variables, summarized as numbers and percentages, were compared using the χ2 test. Continuous variables were expressed as mean and standard deviation (SD) or median and IQR and compared using the Student's t-test. A shared frailty gamma Cox regression with random effects for individual hospitals of enrollment was employed to compare survival according to hospital governance. The model was based on variables such as age, sex, HDIM, per capita income (indices stratified by the mean value of the population of the state of Rio de Janeiro), Gini index (stratified in tertiles), AKI phenotype (community-acquired vs. hospital-acquired, *de novo* vs. ACKD, septic vs. nonseptic AKI, and oliguric vs. nonoliguric AKI), number of failing organs, and Charlson comorbidity score. The results were presented as hazard ratios with a 95% confidence interval. The Schoenfeld residuals analysis was employed to test the assumption of the Cox proportional hazards model. In addition, a separate analysis was conducted that included interaction terms of socioeconomic factors, type of hospital, outcomes, and stratification of hospitals according to the number of beds.

In addition to comparing the entire cohort (14,149 patients in the private group vs. 1037 patients in the public group), a propensity score matching approach was used to create a balanced cohort. A logistic regression model that included demographic and clinical characteristics was employed (Supplementary File S1, section 3, Supplementary Table S1). Propensity-matched cohorts were created using a 1:1 ratio greedy-nearest-neighbor matching on the propensity score and a caliper of 0.1 SD of the propensity score logit with no replacement. The standardized mean differences (SMD) were used to compare covariate balances before and after matching, with successful balancing considered when the SMD was <0.10. To investigate differences between matched groups and possible relationships to outcomes, socioeconomic indicators were not included in the propensity score.

Thirty-day Kaplan-Meier survival curves were constructed for both unmatched and matched cohorts to compare public and private hospitals and HDIM and income. A log-rank test was used to determine the significance of survival differences between curves. In addition to considering individual hospitals as random effects in the mixed model and acknowledging the heterogeneity of medical institutions involved (i.e., the predominance of medium and small private medical facilities), a sensitivity analysis was conducted using only large hospitals under private and public governance. Pearson correlation was employed to compare the associations between HDIM, the Gini index, and per capita income in different hospital governance populations.

## Results

### Characteristics of Private and Public Hospitals and Patient Profiles

We examined data from 15,186 patients with AKI-D collected over an 11-year period (Figure 1). Of the 170 medical institutions involved, 82% (*n* = 139) were under private administration and 18% (*n* = 31) were under public administration. Small-sized and medium-sized general hospitals and medical facilities predominated in the private sector, whereas medium-sized and larger medical institutions predominated in the public sector. Public hospitals had a more diverse specialty distribution, including a higher proportion of emergency or trauma units (Supplementary File S1, section 4, Supplementary Table S2). The distribution of studied hospitals in the cohort within Rio de Janeiro was predominantly concentrated in high-income districts, deviating from the expected pattern of aligning with the most populated communities (Supplementary File S1, section 4, Supplementary Figure S1).

The cohort’s demographic and clinical characteristics are summarized in Table 1 as follows: 14,149 patients were admitted to private hospitals, and 1037 patients were admitted to public hospitals. The median age in private hospitals was 75 years (IQR, 61–83) compared with 53 years (IQR, 31–66) in public hospitals. Private hospitals had a higher proportion of patients who self-declared as of White ethnicity than public hospitals (84.3% vs. 61.0%, *P* < 0.001). Most patients presented with *de novo*-acquired and hospital-acquired AKI-D. However, community-acquired AKI-D was more common in public hospitals (45.7% vs. 27.0%, *P* < 0.001), whereas ACKD disease was more common in private hospitals (33.2% vs. 23%, *P* < 0.001).

**Table 1:** Demographics, clinical characteristics, and outcomes of unmatched and matched cohorts

Variables	All (*N* = 15,186)	Private (*n* = 14,149)	Public (*n* = 1037)	*P* value	SMD	Private (PSM, *n* = 1037)	*P*	SMD
Demographics								
Age (median [IQR])	74 (59–82)	75 (61–83)	53 (31–66)	<0.001	0.965	51(31–68)	0.748	0.023
Male gender (%)	8291 (54.6)	7705 (54.5)	586 (56.5)	0.212	0.041	577 (55.6)	0.723	0.017
Nonwhite (%)	2624 (17.3)	2220 (15.7)	404 (39.0)	<0.001	0.541	383 (36.9)	0.365	0.042
AKI phenotype								
Acute-on-chronic kidney disease (%)	4933 (32.5)	4694 (33.2)	239 (23.0)	<0.001	0.227	240 (23.1)	1.000	0.002
Community-acquired AKI (%)	4293 (28.3)	3819 (27.0)	474 (45.7)	<0.001	0.397	442 (42.6)	0.170	0.062
Medical admission (%)	11397 (75)	10788 (76.2)	609 (58.7)	<0.001	0.381	621 (59.9)	0.623	0.024
ICU admission (%)	13044 (85.9)	12428 (87.8)	616 (59.4)	<0.001	0.682	676 (65.2)	0.008	0.120
Comorbidities								
Chronic heart Diseases (%)	4902 (32.3)	4748 (33.6)	154 (14.9)	<0.001	0.448	168 (16.2)	0.431	0.037
Hypertension (%)	6711 (44.2)	6446 (45.6)	265 (25.6)	<0.001	0.427	277 (26.7)	0.582	0.026
Diabetes (%)	3113 (20.5)	2993 (21.2)	120 (11.6)	<0.001	0.261	130 (12.5)	0.544	0.030
Neoplasia (%)	2227 (14.7)	2010 (14.2)	217 (20.9)	<0.001	0.177	205 (19.8)	0.549	0.029
Chronic neurologic diseases (%)	1536 (10.1)	1490 (10.5)	46 (4.4)	<0.001	0.233	66 (6.4)	0.065	0.085
Obstructive vascular disease (%)	1631 (10.7)	1561 (11.0)	70 (6.8)	<0.001	0.151	54 (5.2)	0.165	0.065
Chronic hepatic Disease (%)	591 (3.9)	565 (4.0)	26 (2.5)	0.021	0.084	24 (2.3)	0.886	0.013
Number of comorbidities (mean [SD])	1.6 (1.16)	1.6 (1.16)	1.1 (1.05)	<0.001	0.516	1.16 (1.02)	0.124	0.068
Charlson score (mean [SD])	2.01 (0.86)	2.03 (0.87)	1.72 (0.75)	<0.001	0.385	1.74 (0.78)	0.421	0.035
Precipitating causes of AKI								
Sepsis at admission (%)	6940 (45.7)	6457 (45.6)	483 (46.6)	0.579	0.019	502 (48.4)	0.429	0.037
Later sepsis (%)	3954 (26.0)	3820 (27.0)	134 (12.9)	<0.001	0.358	144 (13.9)	0.562	0.028
CRS type I (%)	1981 (13.0)	1889 (13.4)	92 (8.9)	<0.001	0.143	116 (11.2)	0.093	0.077
Hypovolemia (%)	4299 (28.3)	3989 (28.2)	310 (29.9)	0.255	0.037	298 (28.7)	0.596	0.025
Nephrotoxicity (%)	1390 (9.2)	1277 (9.0)	113 (10.9)	0.050	0.063	105 (10.1)	0.616	0.025
Urological conditions (%)	736 (4.8)	559 (4.0)	177 (17.1)	<0.001	0.438	150 (14.5)	0.117	0.071
Major surgery (%)	1957 (12.9)	1842 (13.0)	115 (11.1)	0.082	0.059	132 (12.7)	0.278	0.051
CRS type II (%)	650 (4.3)	636 (4.5)	14 (1.4)	<0.001	0.188	13 (1.3)	1.000	0.009
Hepatorenal syndrome (%)	296 (1.9)	287 (2.0)	9 (0.9)	0.013	0.097	11 (1.1)	0.822	0.020
Glomerular diseases (%)	162 (1.1%)	151 (1.1%)	11 (1.1%)	1	0.009	12 (1.2)	1	0.009
Obstetric complications (%)	110 (0.7)	52 (0.4)	58 (5.6)	<0.001	0.163	25 (2.4)	<0.001	0.311
Number of causes (mean [SD])	1.45 (0.62)	1.46 (0.62)	1.42 (0.61)	0.094	0.055	1.47 (0.61)	0.105	0.071
Indications for commencing RRT								
Azotemia (%)	12730 (83.8)	11870 (83.9)	860 (82.9)	0.443	0.026	844 (81.4)	0.390	0.040
Oliguria (%)	11480 (75.6)	10784 (76.2)	696 (67.1)	<0.001	0.203	709 (68.4)	0.573	0.027
Acidosis (%)	11077 (72.9)	10397 (73.5)	680 (65.6)	<0.001	0.172	700 (67.5)	0.377	0.041
Hypervolemia (%)	5688 (37.5)	5288 (37.4)	400 (38.6)	0.461	0.025	416 (40.1)	0.500	0.032
Hyperkalemia (%)	3125 (20.6)	2758 (19.5)	367 (35.4)	<0.001	0.362	329 (31.7)	0.085	0.078
Number of indications (mean (SD))	2.92 (1.02)	2.92 (1.01)	2.94 (1.20)	0.508	0.020	2.94 (1.11)	0.924	0.004
Organ failures								
Mechanical ventilation (%)	11424 (75.2)	10823 (76.5)	601 (58.0)	<0.001	0.403	646 (62.3)	0.048	0.089
Vasopressors (%)	10829 (71.3)	10305 (72.8)	524 (50.5)	<0.001	0.471	574 (55.4)	0.031	0.097
Isolated kidney failure (%)	2655 (17.5)	2370 (16.8)	288 (27.5)	<0.001	0.368	257 (24.8)	0.255	0.089
Additional failing organs (mean (SD))	2.03 (1.23)	2.06 (1.21)	1.66 (1.32)	<0.001	0.312	1.78 (1.32)	0.035	0.093
RRT modality (%)				<0.001	0.651		0.249	0.089
PIRRT	7112 (46.8)	6909 (48.8)	203 (19.6)			204 (19.7)		
IHD	4859 (32.0)	4343 (30.7)	516 (49.8)			477 (46.0)		
C-PIRRT	2890 (19.0)	2612 (18.5)	278 (26.8)			305 (29.4)		
PD	325 (2.1)	285 (2.0)	40 (3.9)			51 (4.9)		
Hospital stays, days (median [IQR])	21 (11–29)	21 (11–39)	17 (8–32)	<0.001	0.171	16 (9–32)	0.946	0.033
Days in RRT (median [IQR]	10 (4-23)	10 (4-23)	9 (3-30)	<0.001	0.187	10 (4-20)	0.193	0.013
Mortality (%)	10771 (70.9)	10154 (71.8)	617 (59.5)	<0.001	0.353	631 (60.8)	0.597	0.073

AKI, acute kidney injury; C-PIRRT, continuous mode PIRRT; CRS, cardiorenal syndrome; ICU, intensive care unit; IHD, intermittent hemodialysis; IQR, interquartile range; PD, peritoneal dialysis; PIRRT, prolonged intermittent renal replacement therapy; PSM, propensity score matching; RRT, renal replacement therapy; SMD, standardized mean difference.

**Figure 2:**
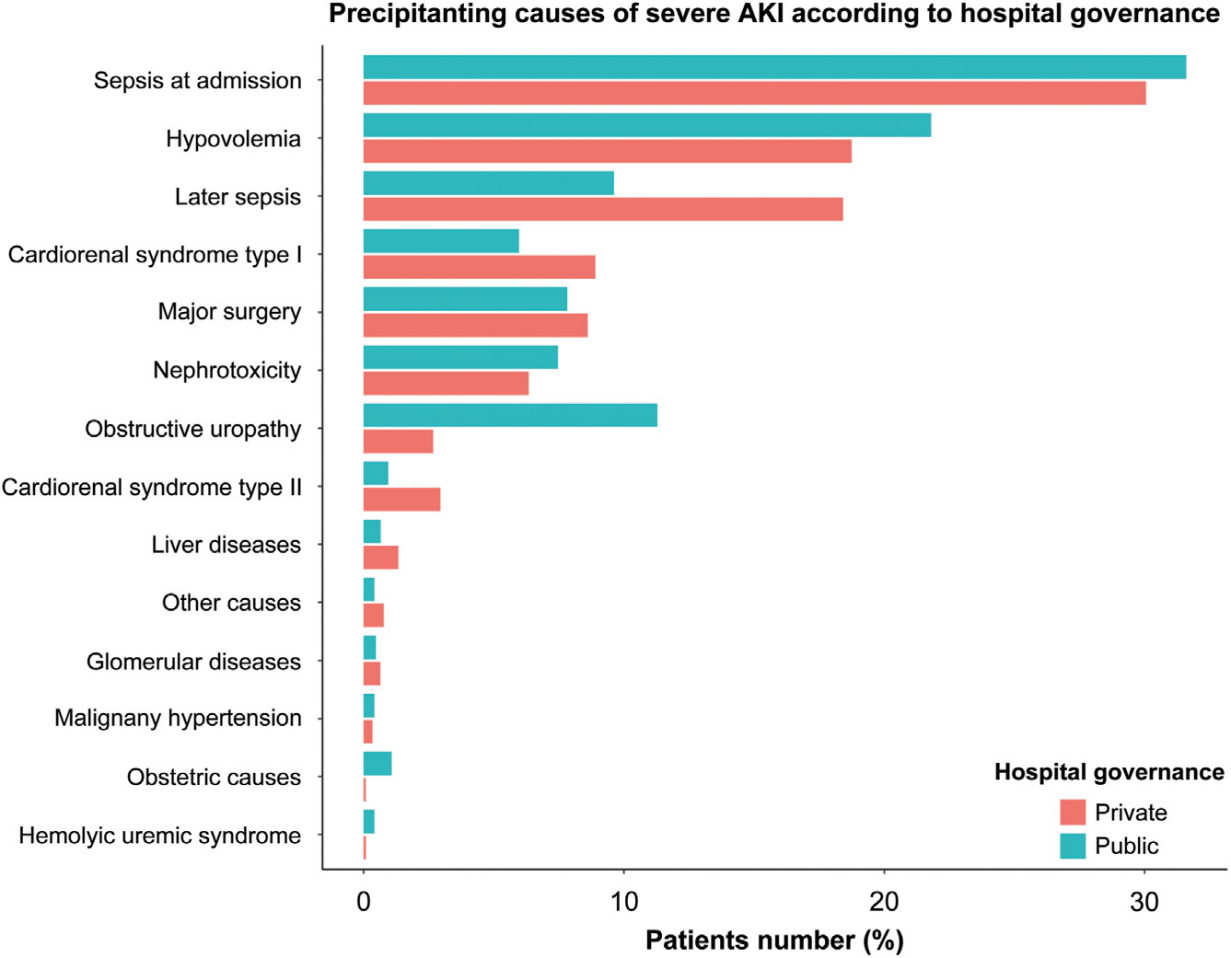
Precipitant causes of severe dialysis-requiring acute kidney injury stratified by hospital governance. Causes are not mutually exclusive.

Comorbidities were more prevalent in private hospitals, except for malignancy (Table 1). The Charlson comorbidity score (2.03 ± 0.87 vs. 1.72 ± 0.75, *P* < 0.001) was higher in private hospitals. Medical reasons for admission and intensive care unit admission predominated in private hospitals (*P* < 0.001). Incidence of sepsis at admission, major surgery, nephrotoxicity, and glomerular diseases as AKI-D causes were similar. Private hospitals had a higher rate of sepsis after admission, cardiorenal syndrome type I and II, and hepatorenal syndrome. Hypovolemia, obstructive uropathy, and preeclampsia were more common in public hospitals (Figure 2). Mechanical ventilation and vasopressor support were used more in private hospitals (*P* < 0.001). Isolated kidney failure was more common in public hospitals (27.5% vs. 16.8%, *P* < 0.001).

Most patients were treated with PIRRT in private hospitals, whereas intermittent hemodialysis was the most common initial RRT modality in public hospitals (Table 1). However, the use of PIRRT in continuous mode was greater in public hospitals than in private hospitals. Finally, the length of stay in private hospitals was significantly longer (21 days, IQR: 11–39) than in public hospitals (17 days, IQR: 8–32).

Each group received 1037 cases after propensity score matching (Table 1). The matching process resulted in a well-balanced cohort. Most baseline-matched covariates showed a decrease of <0.1 in SMD between private and public hospitals, except for socioeconomic covariates, which were purposefully excluded from the matching process. Baseline demographics, AKI-D phenotype, comorbidities, AKI-D precipitating causes (except obstetric conditions) and RRT modalities were comparable.

### Socioeconomic Disparities in Private and Public Hospital Admissions

**Figure 3:**
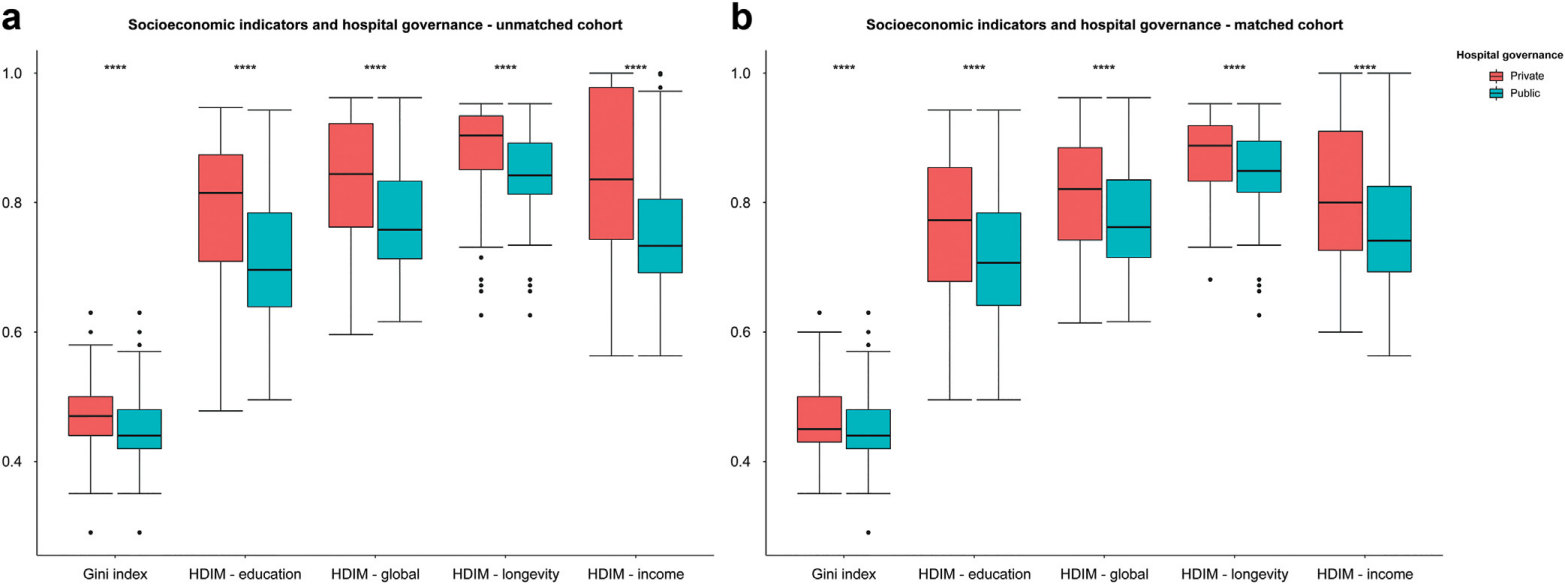
Socioeconomic indicators of patients with dialysis-requiring acute kidney injury among private versus public hospitals. (a) unmatched and (b) matched cohorts. HDIM, human development index (municipality).

**Figure 4:**
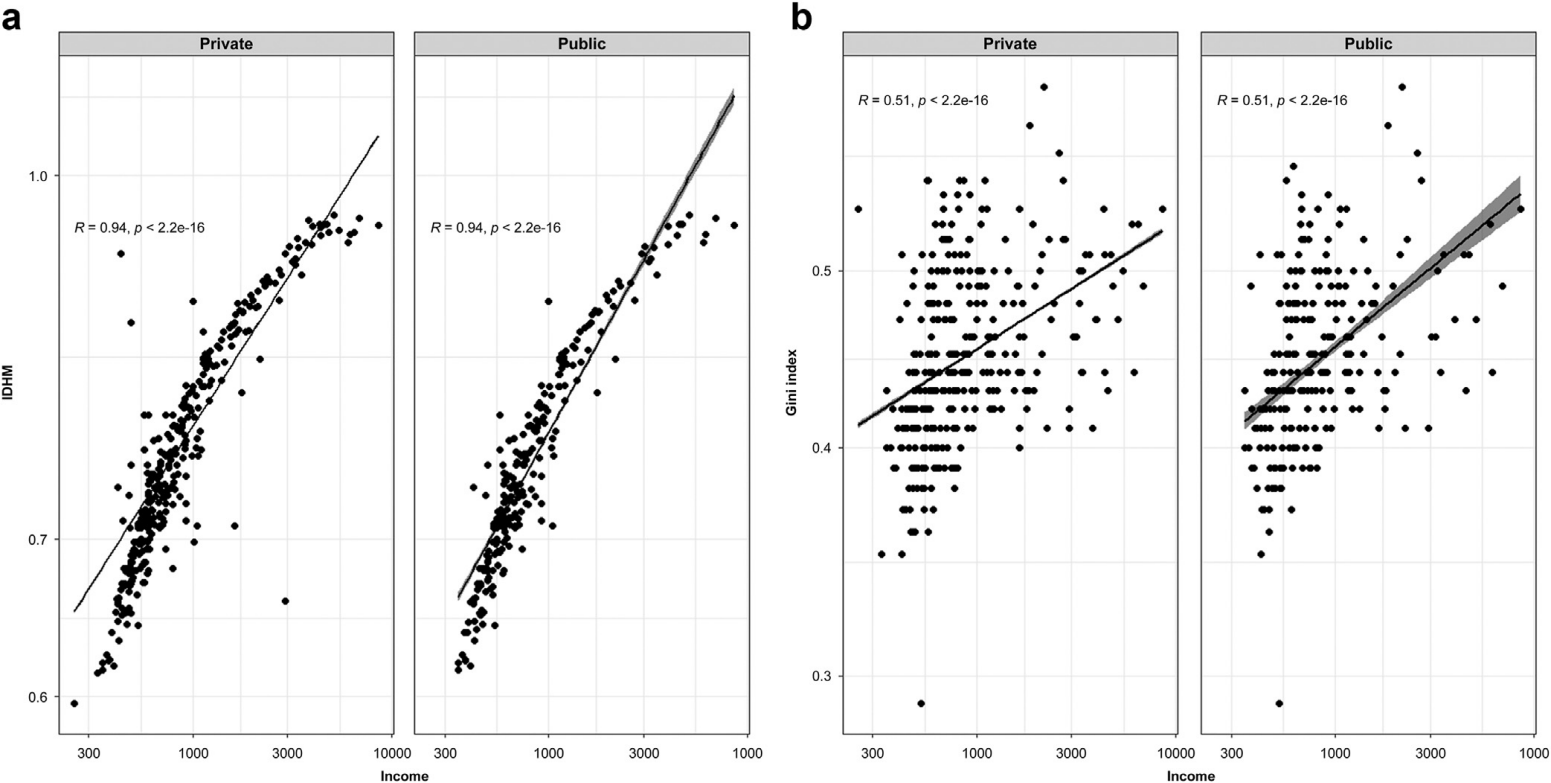
Correlation between income and HDIM and Gini index according to hospital administration. HDIM: human development index (municipality). Income is presented in Brazilian currency (Reais, R$). HDIM, human development index (municipality).

The socioeconomic indicators differed significantly between patients in private and public hospitals (Table 2 and Figure 3). This pattern persisted after the matching process. The HDIM was significantly higher in private hospitals than in public hospitals, and this difference was consistent across all HDIM index subdivisions. A lower Gini index distinguished patients in public hospitals from private hospitals. Income was significantly higher in private hospitals (Table 2). Regardless of hospital governance, there was a significant relationship between socioeconomic indicators and income level as follows: higher incomes were associated with higher HDIM (R = 0.94, *P* < 0.001) and greater inequality (measured by the Gini index) (R = 0.51, *P* < 0.001), in both private and public hospitals (Figure 4). All socioeconomic indicators were lower among non-White patients than among White patients, but this difference was only significantly different after stratification in private hospital patients (Supplementary File S1, section 5, Supplementary Tables S3 and S4, Supplementary Figure S2).

**Table 2:** Socioeconomic indicators according to hospital governance in the unmatched and matched cohorts

Variables	All (n = 15186)	Private (n = 14149)	Public (n = 1037)	*P*	SMD	Private (PSM, n = 1037)	*P* value	SMD
Socioeconomic indicators								
HDIM - Global (mean [SD])	0.83 (0.09)	0.84 (0.09)	0.78 (0.08)	<0.001	0.732	0.82 (0.09)	<0.001	0.494
HDIM - Income (mean [SD])	0.84 (0.12)	0.84 (0.12)	0.76 (0.09)	<0.001	0.742	0.82 (0.11)	<0.001	0.501
HDIM - Longevity (mean [SD])	0.88 (0.05)	0.89 (0.05)	0.85 (0.05)	<0.001	0.684	0.88 (0.05)	<0.001	0.478
HDIM - Education (mean [SD])	0.78 (0.10)	0.79 (0.10)	0.72 (0.09)	<0.001	0.692	0.76 (0.11)	<0.001	0.462
Income (mean [SD])	2059.95 (1642.21)	2122.61 (1658.97)	1205.01 (1076.27)	<0.001	0.656	1810.67 (1560.05)	<0.001	0.452
Gini Index (mean [SD])	0.47 (0.05)	0.47 (0.05)	0.46 (0.05)	<0.001	0.281	0.47 (0.06)	<0.001	0.200

HDIM, human development index (municipality); PSM, propensity score matching; SMD, standardized mean difference.

### Mortality and Discharge Outcomes

**Figure 5:**
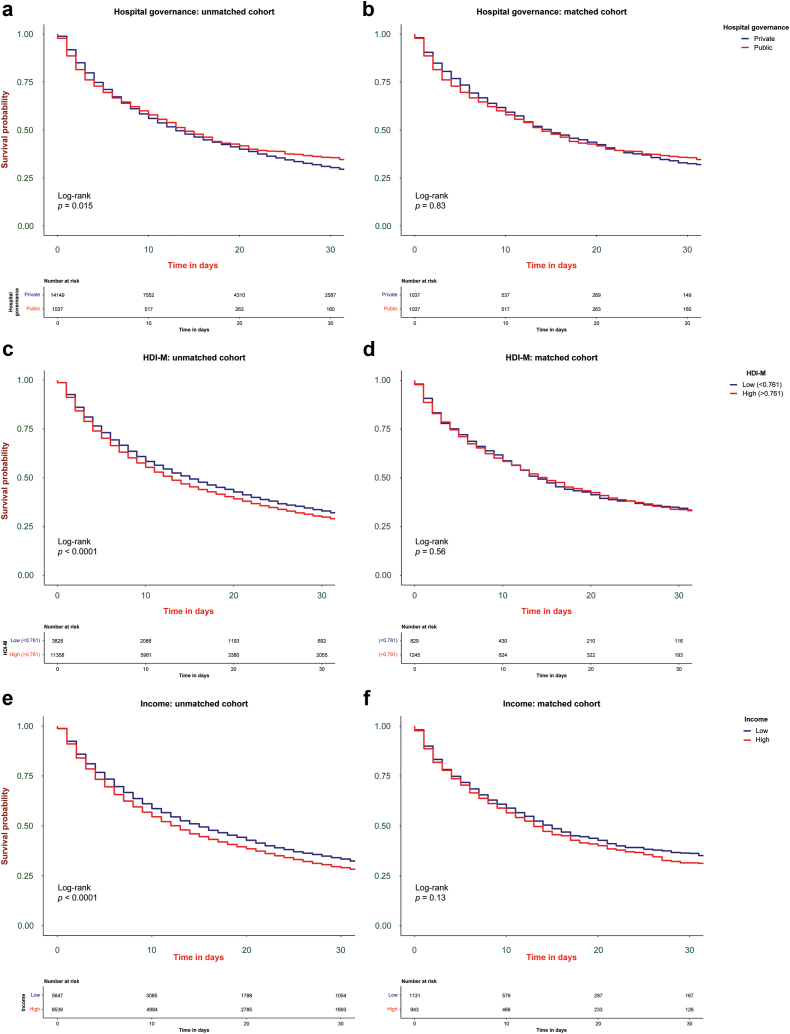
Survival curves, according to Kaplan–Meier estimates according to hospital administration (a and b), HDIM (c and d), and income (e and f), with log-rank test for significance, in the unmatched and matched cohorts. HDIM: human development index (municipality).

Within 30 days after RRT initiation, the crude mortality rate was 70.9% (10,771 patients), with the private group accounting for 71.8% (10,154 patients) and the public group accounting for 59.5% (617 patients, *P* < 0.001). In contrast, mortality rates in the balanced matched cohort were comparable (60.8% vs. 59.5% in private vs. public hospitals, *P* = 0.597). The median length of hospital stay was higher in private hospitals (21 days, IQR: 11–39) compared to public hospitals (18 days, IQR: 8–32.75, *P* < 0.001). The Kaplan–Meier curves for overall mortality in the unmatched and matched cohorts are shown in Figure 5a and 5b. Worse socioeconomic indicators (HDIM, income, and Gini index) in the univariate mixed-effects Cox proportional model of the unmatched cohort were all associated with higher mortality (Table 3). This difference remained significant only for higher income in the multivariate model. However, no socioeconomic variable was significantly associated with mortality in the multivariate model (Figure 5c–f).

**Table 3:** Univariate and multivariate mixed-effects Cox regression model on mortality in the matched and unmatched cohort

Variables	HR (univariable)	HR (multivariable)	HR (univariable)	HR (multivariate)
	Full Cohort (*N* = 15,186 patients)		Matched Cohort (*n* = 2074 patients)	
Age				
(per yr)	1.01 (1.01–1.01, *P* < 0.001)	1.01 (1.01–1.01, *P* < 0.001)	1.01 (1.01–1.01, *P* < 0.001)	1.01 (1.01–1.01, *P* <0.001)
Gender	-	-	-	-
Female				
Male	0.88 (0.85–0.92, *P* < 0.001)	0.92 (0.89–0.96, *P* < 0.001)	1.07 (0.96–1.20, *P* = 0.238)	0.98 (0.87–1.10, *P* = 0.717)
Ethnicity	-	-	-	-
White				
Nonwhite	0.94 (0.90–0.99, *P* = 0.027)	1.00 (0.95–1.06, *P* = 0.975)	1.02 (0.91–1.14, *P* = 0.751)	1.09 (0.97–1.23, *P* = 0.148)
Hospital Governance	-	-	-	-
Private				
Public	0.91 (0.83–0.98, *P* = 0.017)	0.98 (0.82–1.17, *P* = 0.852)	0.99 (0.89–1.11, *P* = 0.852)	0.92 (0.76–1.11, *P* = 0.380)
AKI phenotype 1	-	-	-	-
Acute				
ACKD	0.75 (0.72–0.78, *P* < 0.001)	0.85 (0.81–0.89, *P* < 0.001)	0.72 (0.63–0.83, *P* < 0.001)	0.89 (0.76–1.03, *P* = 0.125)
AKI phenotype 2	-	-	-	-
Hospital-acquired				
Community-acquired	0.65 (0.63–0.69, *P* < 0.001)	0.86 (0.82–0.90, *P* < 0.001)	0.59 (0.52–0.66, *P* < 0.001)	0.86 (0.75–0.98, *P* = 0.021)
AKI phenotype 3	-	-	-	-
Nonseptic AKI				
Septic AKI	1.28 (1.24–1.33, *P* < 0.001)	1.14 (1.09–1.18, *P* < 0.001)	1.65 (1.47–1.84, *P* < 0.001)	1.36 (1.21–1.53, *P* < 0.001)
AKI phenotype 4	-	-	-	-
Nonoliguric AKI				
Oliguric AKI	1.55 (1.48–1.63, *P* < 0.001)	1.32 (1.25–1.38, *P* < 0.001)	1.76 (1.55–2.00, *P* < 0.001)	1.35 (1.17–1.55, *P* < 0.001)
Additional organ failure				
(per organ)	1.37 (1.35–1.40, *P* < 0.001)	1.34 (1.32–1.37, *P* < 0.001)	1.45 (1.40–1.52, *P* < 0.001)	1.39 (1.32–1.46, *P* < 0.001)
Charlson score				
(per point)	1.02 (1.00–1.04, *P* = 0.062)	1.00 (0.97–1.02, *P* = 0.707)	1.05 (0.98–1.13, *P* = 0.183)	1.01 (0.94–1.10, *P* = 0.727)
HDIM	-	-	-	-
Low				
High	1.10 (1.05–1.15, *P* < 0.001)	0.98 (0.92–1.05, *P* = 0.615)	1.03 (0.92–1.16, *P* = 0.568)	0.89 (0.75–1.06, *P* = 0.189)
Income	-	-	-	-
Low				
High	1.13 (1.09–1.18, *P* < 0.001)	1.08 (1.01–1.15, *P* = 0.025)	1.11 (0.99–1.24, *P* = 0.068)	1.18 (1.00–1.41, *P* = 0.054)
Gini Index	-	-	-	-
0.29 to 0.44				
0.44 to 0.48	1.05 (1.00–1.09, *P* = 0.062)	1.01 (0.96–1.06, *P* = 0.789)	1.07 (0.94–1.23, *P* = 0.293)	1.10 (0.95–1.26, *P* = 0.198)
0.48 to 0.63	1.10 (1.05–1.15, *P* < 0.001)	0.98 (0.93–1.04, *P* = 0.567)	1.17 (1.02–1.34, *P* = 0.026)	1.15 (0.99–1.34, *P* = 0.073)

AKI, acute kidney injury; ACKD, acute-on-chronic kidney disease; HR, hazard ratio; HDIM, human development index (municipality)

Multivariate analysis in the unmatched and matched cohorts showed that age, oliguric AKI, septic AKI, and the number of organ failures were all independently associated with mortality. In the unmatched cohort, male gender, community-acquired AKI-D, and ACKD were associated with better survival (Table 3).

Among the 4415 survivors (3995 in private hospitals and 420 in public hospitals), 1034 (23.4%) were discharged on RRT (945 [23.7%] vs. 89 [21.2%] in private vs. public hospitals [*P* < 0.001]). Discharge with complete recovery was higher in private hospitals (27.8%) than in public hospitals (16%), whereas discharge with partial recovery was higher in public hospitals (49% vs. 30.3%). The kidney status at discharge was unknown in 17.8% of the patients. The discharge outcomes did not differ based on socioeconomic indicators (Supplementary File S1, section 7, Supplementary Table S7).

### Sensitivity Analysis Results

A sensitivity analysis of larger hospitals (20 private vs. 21 public, 6993 patients) revealed no significant differences in patient characteristics and outcomes compared to the entire cohort (Supplementary File S1, section 6, Supplementary Table S4 and S5). Comparing the original mixed-effect Cox model with the model including additional interaction terms and hospital stratification by the number of beds showed no significant improvement in performance (Supplementary File S1, section 6, Supplementary Table S6).

## Discussion

Our study aims to investigate the impact of socioeconomic indicators on outcomes among patients with AKI. This topic has only been explored in high-income countries using large administrative databases. In addition, our study is the first to compare the outcomes of patients with AKI-D in private versus public hospital settings. Although previous studies have examined the incidence of AKI in different hospital administrations as a complication after various medical procedures,^17^ none have specifically investigated the epidemiology of AKI in these settings.

Age, sepsis, oliguria, and the number of failing organs were associated with poor survival, whereas male versus female gender, community-acquired versus hospital-acquired AKI, and ACKD versus *de novo* AKI had better outcomes. These clinical characteristics and associations are well documented in other comprehensive series of AKI publications.^18^, ^19^, ^20^, ^21^, ^22^, ^23^, ^24^, ^25^ Interestingly, in the context of the present study, some of these covariates were unequally distributed between patients assisted in private versus public hospitals.

Previous research in Brazilian intensive care units compared sepsis outcomes in patients admitted to public versus private hospitals, with contradictory results. One early multicenter study using a convenience sample of patients with sepsis found that those admitted to public hospitals fared worse than those treated in private hospitals. In contrast, a more extensive and possibly less biased multicenter study on a large stratified pseudo-random sample of healthcare institutions found no difference in mortality between inpatients with sepsis admitted to public or private intensive care units.^13^^,^^26^ In the present study, which only included patients with AKI-D, crude mortality was higher in private hospitals. However, after adjusting for confounding, mortality rates were not significantly different, particularly in the propensity-matched cohort, indicating that, at least in the particular environment under study, other demographic and clinical features would appear to be the main drivers of poor outcomes in AKI-D.

The socioeconomic indicators varied depending on the hospital administration. Higher socioeconomic indicators were associated with higher mortality in the unadjusted analysis, a pattern that was also observed in other population-level analyses.^5^^,^^8^ However, after adjustments, there was no significant impact of HDIM, its components (education, income, and longevity), and the Gini coefficient on survival. Higher income was associated with poorer survival, but other unmeasured factors may have influenced this, because high-income patients were primarily treated in private hospitals. The median age was also significantly higher among private hospital patients. An essential aspect of aging that was not specifically quantified is the increasing prevalence of frailty, which is associated with worse outcomes. Our data suggest that the higher mortality in higher-income patients predominantly treated in private hospitals may reflect the unmeasured frailty component of the aging process.^8^ It has already been demonstrated that crude mortality rates following AKI are higher in high-income areas, indicating an older patient population.^5^ Notably, this relationship was not observed in the multivariate model of the balanced matched cohort, where the median age was considerably lower. Finally, our findings differ from those in high-income countries, where the burden of comorbidity and the severity of AKI at presentation may contribute to the impact of social deprivation on AKI-associated mortality.^5^^,^^8^^,^^10^

Despite significant differences in socioeconomic indicators related to ethnicity and a significant difference in the distribution of these groups in the 2 hospital systems, we found no independent influence of race on survival, contrary to findings in high-income countries.^27^ More significant economic deprivation and a higher burden of comorbidities in the non-White population are characteristics of social discrepancies in Brazil,^28^ but they did not translate into a higher mortality risk in our study.

Sepsis at admission was the most common condition associated with AKI-D development in private and public hospitals, according to several cohort studies of AKI.^6^^,^^19^^,^^20^^,^^23^ However, markers of poorer outcomes, such as sepsis after admission, cardiorenal syndrome type I, and hepatorenal syndrome, were more common in private hospitals, whereas factors associated with better survival odds, such as obstructive uropathy and preeclampsia, were more common in public hospitals. This difference partly explains the lower survival observed in private hospitals.

We found no association between socioeconomic indicators and renal recovery among survivors. This finding differs from an epidemiologic study conducted in Taiwan, which identified higher regional economic status as an independent factor for renal recovery.^6^ Our study revealed a high prevalence of patients discharged with partial renal recovery (33.03%) or receiving RRT (23.3%). Interestingly, more patients in private hospitals were discharged on RRT compared to public hospitals (23.7% vs. 21.2%). Our previous publications with the same cohort showed a significant association between preexisting CKD and these adverse kidney outcomes.^15^ The higher median age of the cohort may partly explain these findings. However, we acknowledge that the lack of long-term follow-up of the patients may have affected our analysis of kidney recovery outcomes.

Our findings must be interpreted in the context of the study's limitations. First, the distribution of private and public hospitals in this cohort does not reflect the actual situation of the Brazilian healthcare system. Approximately 90% of inpatients in this cohort were admitted to private hospitals, even though only 20% to 25% of the Brazilian population has private health insurance.^27^ Furthermore, it was not our intention to produce a population-level analysis regarding the incidence and prevalence of AKI-D. As stated, the institutions involved do not represent the whole scope of Rio de Janeiro’s medical facilities, as can be gathered by the higher prevalence of private hospitals in the cohort.^15^ It is also important to note that the hospitals studied in the manuscript were unequally distributed in the city of Rio de Janeiro, showing higher concentrations in neighborhoods with higher human development index and not according to population distribution. This uneven distribution of hospitals may be indicative of existing inequities in the accessibility and availability of healthcare services within the city, which could impact the generalizability of our findings. Second, significant differences were observed in the phenotypes of hospitals, with small or medium medical institutions in the private sector outnumbering large hospitals in the public sector. Therefore, we conducted a sensitivity analysis limited to larger hospitals from both types of governance, which reproduced the results of the entire cohort. Third, given the various health determinants and potential disparities in care quality across our county, these findings may not be extrapolated to all regions of the vast Brazilian territory.^28^ Fourth, socioeconomic data were retrieved from census information using the residence code rather than individual-level measures of socioeconomic status, which may have resulted in some misclassification.^10^ However, this strategy is mainly used in studies that assess the socioeconomic status and AKI incidence and outcomes.^5^^,^^8^^,^^10^ Finally, this was a short-term follow-up study, from admission to discharge, and we were not able to follow-up with these patients in the long-term to account for outcomes among survivors. Patients were discharged either with complete or partial recovery or with dialysis dependence. We acknowledge that some AKI survivors discharged on RRT may eventually become dialysis independent during the follow-up period, and unfortunately, we were not able to capture this important information.^29^

### Conclusion

AKI-D has short-term and long-term adverse consequences. Despite differences in socioeconomic characteristics, the present study found no impact of different hospital governance systems on overall mortality. Poor AKI outcomes were primarily driven by the patient's baseline characteristics and the severity of the presentation. Future large-scale epidemiologic studies should further investigate these relevant assumptions, allowing healthcare systems to manage this severe syndrome promptly and accurately.

## Disclosure

FR and JHRS are minor partners of KMR2, a spinoff company of Kidney Assistance Ltd that developed the NefroWeb product to manage logistics and billing related to dialysis services in hospitals and clinics in the metropolitan area of Rio de Janeiro. JHRS conceived NefroWeb’s clinical module solely for epidemiologic research purposes, with no financial relationship that could appear to have influenced the submitted work. The 2 other authors have no affiliation to KMR2 and no conflicting interests to disclose.

## Supplementary Material

Supplementary File (PDF)

**File S1.** About the NefroWeb database. Data acquisition, categorizations, indication of dialysis, organ failure definition, and dialysis modalities.

**Figure S1.** Spatial distribution of medical institutions in Rio de Janeiro City, according to (a) population and (b) HDIM.

**Figure S2.** Socioeconomic distribution stratified by ethnicity sensitivity analysis–large hospitals of private and public governance.

**Table S1.** Variables used in initial logistic regression for the propensity score matching process involved medical institutions

**Table S2.** Distribution of size and specialty of enrolled medical institutions Socioeconomic distribution stratified by ethnicity.

**Table S3.** Socioeconomic distribution stratified by ethnicity.

**Table S4.** Comparison of large hospitals of private vs. public governance.

**Table S5.** Survival according to a frailty gamma Cox regression in the large hospitals’ cohort.

**Table S6.** Addition of an interaction term (socioeconomic factors ∗ hospital type ∗ outcomes) and number of beds in the model.

**Table S7.** Discharge outcomes among survivors, stratified by hospital administration and socioeconomic indicators.
