# Supplementary material for: Socioeconomic Influences on the Outcomes of Dialysis-Requiring Acute Kidney Injury in Brazil
Source: Kidney Int Rep. 2023 Jun 14;8(9):1772–83. doi: 10.1016/j.ekir.2023.06.003 (PMC10496017; doi:10.1016/j.ekir.2023.06.003)
Supplement: Supplementary File (PDF) [file mmc1.pdf]

Supplemental File S1

Socioeconomic Influences on the Outcomes of Dialysis-Requiring Acute Kidney Injury in the Two-Tiered Healthcare System of Brazil: A Retrospective Cohort Study

Conrado Lysandro R. Gomes, Thais Lyra Cleto Yamane, Frederico Ruzani, José Hermógenes Rocco Suassuna

INDEX

1. About the NefroWeb database
2. Data acquisition, categorizations, indication of dialysis, organ failure definition and dialysis modalities.
3. Table S1. Variables used in initial logistic regression for the propensity score matching process
4. Involved medical institutions
    4.1 Table S2. Distribution of size and specialty of enrolled medical institutions
    4.2 Figure S1. Spatial distribution of medical institutions in Rio de Janeiro City, according to (a) population and (b) HDIM.
5. Socioeconomic distribution stratified by ethnicity
    5.1 Table S3. Socioeconomic distribution stratified by ethnicity
    5.2 Figure S2. Socioeconomic distribution stratified by ethnicity
6. Sensitive analysis – Large hospitals of private and public governance
    6.1 Table S4. Comparison of large hospitals of private vs. public governance
    6.2 Table S5. Survival according to a frailty gamma Cox regression in the large hospitals' cohort
    6.3 Table S6. Addition of an interaction term (socioeconomic factors*hospital type*outcomes) and number of beds in the model
7. Table S7. Discharge outcomes among survivors, stratified by hospital administration and socioeconomic indicators.

APPENDEX

1. STROBE Statement

1. **The NefroWeb® database**

The web-based NefroWeb® is a relational database developed for administrative purposes using the Microsoft SQL Server management system. It was designed to support a privately outsourced mobile inpatient dialysis enterprise in a wide range of public and private hospitals in the metropolitan area of Rio de Janeiro, Brazil. A clinical and epidemiological module was later added to the original database.

Each attending nephrologist was assigned a unique user ID and password to gain access to the database. The individual record of each incident patient contained all the variables described in the Methods section, along with a structured written report. RRT initiation date corresponded to the time zero, which was used as the primary reference for the study. The records were anonymized, exported to Microsoft Excel, and individually reviewed for consistency and categorization of variables before exporting for statistical analysis.

After the preliminary analysis and database expansion described in the Methods section, every new record included checklists containing at least one primary diagnostic group, one renal diagnosis, an extensive choice of comorbidities, and clinical setting details. This uniformity of date collections implies that, despite the potential differences in hospital complexity and specialization, staffing, number of beds, access to state-of-the-art technology, and overall quality of care, the final database was remarkably consistent.

2. **Data acquisition, categorizations, indication of dialysis, organ failure definition and dialysis modalities.**

The attending nephrologist was responsible for patient evaluation, indication of dialysis and the appropriate method, and collection of relevant data for feeding the database. We conducted these procedures on the day of dialysis indication by reviewing medical reports, physical examination, and laboratory results. The collection of a considerable amount of data, including raw data, would substantially increase the medical practitioner's daily burden. Therefore, we dichotomized most of our data into categories, including comorbidities and the presence of preexisting CKD (data imputed by attending nephrologist according to medical history, examination of preexisting laboratory results, if available, and caregiver consultation). The recording of organ failures, for instance, was also a process based on clinical judgment, onsite laboratory review, and current medical practice and knowledge, resulting in the categorization of organ failure and SOFA score computing. According to classical indications (e.g., refractory hyperkalemia, hypervolemia, severe acidemia, azotemia) or clinical judgment (e.g., progressive fluid overload), the initiation of dialysis was at the discretion of the attending nephrologist. The coding of precipitating causes of AKI was also based on clinical judgment at the bedside, medical history and chart review, physical examination, laboratory tests, and image studies mirroring the real-life aspects of nephrology actuation. Based on clinical judgment and laboratory review, this sort of medical evaluation was also applied for other categorizations, such as the definitions of precipitating cause of AKI (e.g., sepsis, hypovolemia, CRS type I).

Dialysis modalities included continuous automated peritoneal dialysis and haemodialysis with standard machinery in following methods: conventional intermittent dialysis, prolonged intermittent renal replacement therapy (PIRRT), or PIRRT in continuous mode (C-PIRRT, meaning continuous haemodialysis provided with standard dialysis equipment and PIRRT set-up parameters). In our part of the world, continuous renal replacement

therapy (CRRT) is 3 to 6 times more expensive than PIRRT or C-PIRRT, and many healthcare providers do not pay for it. Also, during the study period, our outsourcing dialysis company that attended the institutions that comprised the cohort had not yet sought approval from the country’s regulatory authorities to provide CRRT. Therefore, volume overloaded or hemodynamically unstable patients that required RRT were treated with PIRRT or C-PIRRT. Of note, only a few other medical institutions in Rio de Janeiro performed CRRT with equipment provided by a different dialysis company during that period.

Of note, no compelling evidence for the superiority of one mode of RRT above the other has been provided, and KDIGO guidelines suggest that the choice of RRT modality must be tailored to the available resources of the attending institution.

3. Table S1. Variables used in initial logistic regression for the propensity score matching process

Age	Number of causes
Gender	Medical admission
Ethnicity	Surgical admission
AKI *de novo* vs. Acute-on-chronic kidney disease	Obstetric admission
Community-acquired vs. Hospital-acquired	Urological conditions
Arterial hypertension	ICU admission
Diabetes	Sepsis at admission
Chronic heart disease	Later sepsis
Neoplasia	Cardiorenal syndrome type I
Chronic hepatic disease	Nephrotoxicity
Charlson score	Hepatorenal syndrome
Hypovolemia	Mechanical ventilation
Metabolic acidosis	Vasopressors
Oliguria	Number of failing organs
Hyperkalemia	Type of renal replacement therapy
Uremia	

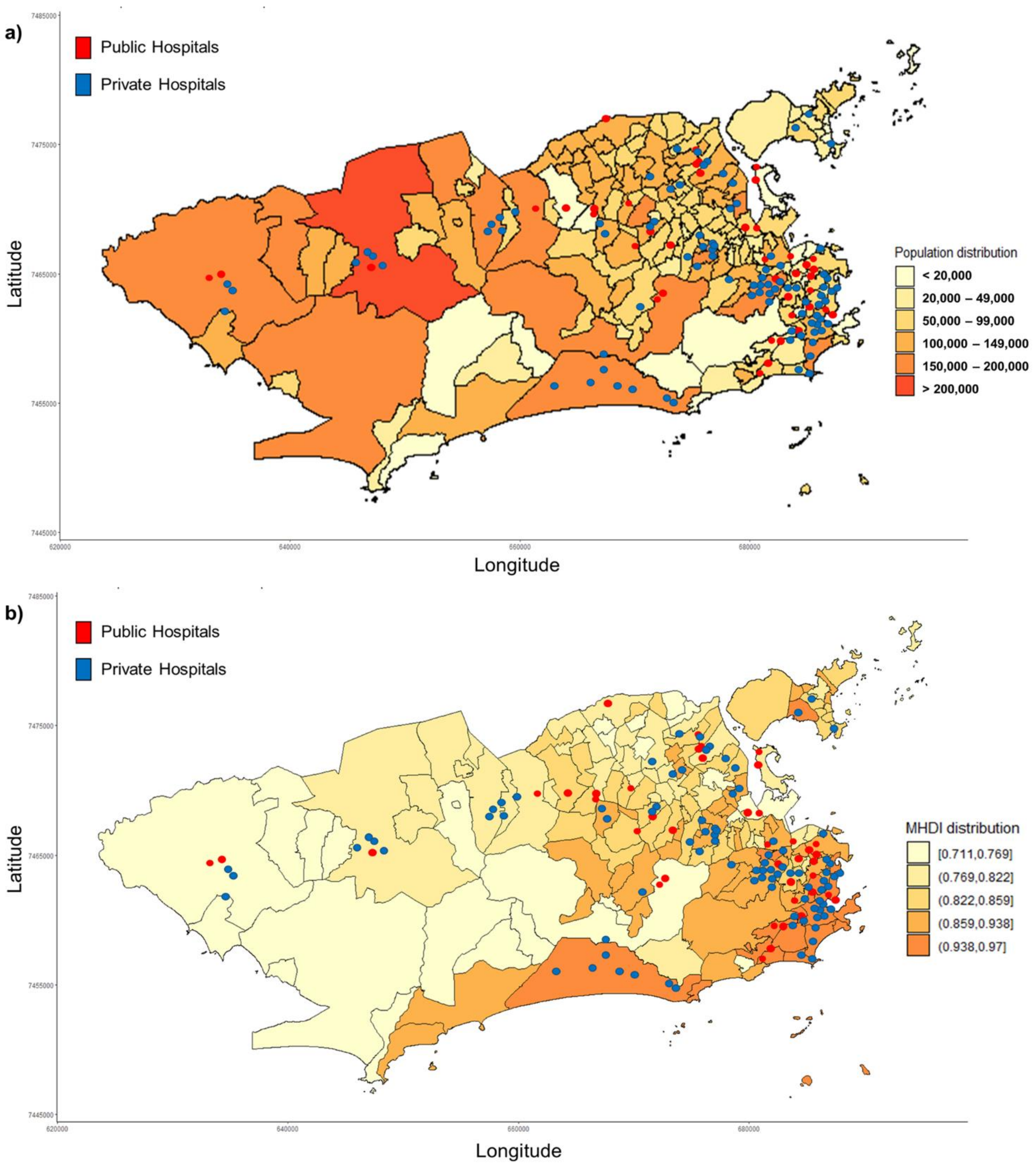

Figure S1. Spatial distribution of medical institutions in Rio de Janeiro City, according to (a) population and (b) HDIM.

4. Involved medical institutions

During the 11-year period, our medical group provided high-quality nephrological support for a total of 170 medical institutions in the metropolitan region of Rio de Janeiro. There was a predominance of private hospitals and medical facilities that corresponded to 82% of total institutions (n = 139 private institutions), and the representativity of public

institutions (18%, n = 31 public institutions) was lower than the actual distribution in Rio de Janeiro. Table S2 depicts the characteristics of involved institutions.

4.1 Table S2. Distribution of size and speciality of enrolled medical institutions.

	All (n = 170)	Private (n = 139; 81.8%)	Public (n = 31; 18.2%)
**Size (number of beds)**			
Small (<50)	57 (33.5%)	57 (41%)	0 (0%)
Medium (51–150)	72 (42.4%)	62 (44.6%)	10 (32.3%)
Large (>151)	41 (24.1%)	20 (14.4%)	21 (67.7%)
**Specialty**			
General	119 (70.0%)	106 (76.3%)	13 (41.9%)
Pediatric	14 (8.2%)	11 (7.9%)	3 (9.7%)
Cardiology	13 (7.6%)	11 (7.9%)	2 (6.5%)
Emergency/Trauma	11 (6.5%)	3 (2.2%)	8 (25.8%)
Obstetric	8 (4.7%)	7 (5.0%)	1 (3.2%)
Oncology	3 (1.8%)	1 (0.7%)	2 (6.5%)
Infectious Diseases	2 (1.2%)	0 (0%)	2 (6.5%)

5.1 Table S3. Socioeconomic distribution stratified by ethnicity

	Overall	White	Nonwhite	p
n	15186	12536	2617	
Gini Index (mean (SD))	0.47 (0.05)	0.47 (0.05)	0.46 (0.05)	<0.001
HDIM - Global (mean (SD))	0.83 (0.09)	0.84 (0.09)	0.80 (0.08)	<0.001
HDIM - Income (mean (SD))	0.84 (0.12)	0.85 (0.12)	0.79 (0.10)	<0.001
HDIM - Longevity (mean (SD))	0.88 (0.05)	0.89 (0.05)	0.86 (0.05)	<0.001
HDIM - Education (mean (SD))	0.78 (0.11)	0.79 (0.11)	0.74 (0.10)	<0.001
Income (R$) (mean (SD))	2099.11 (1674.56)	2242.14 (1719.69)	1408.34 (1213.58)	<0.001

SD: standard deviation; HDIM: human development index (municipality).

5.2 Figure S2

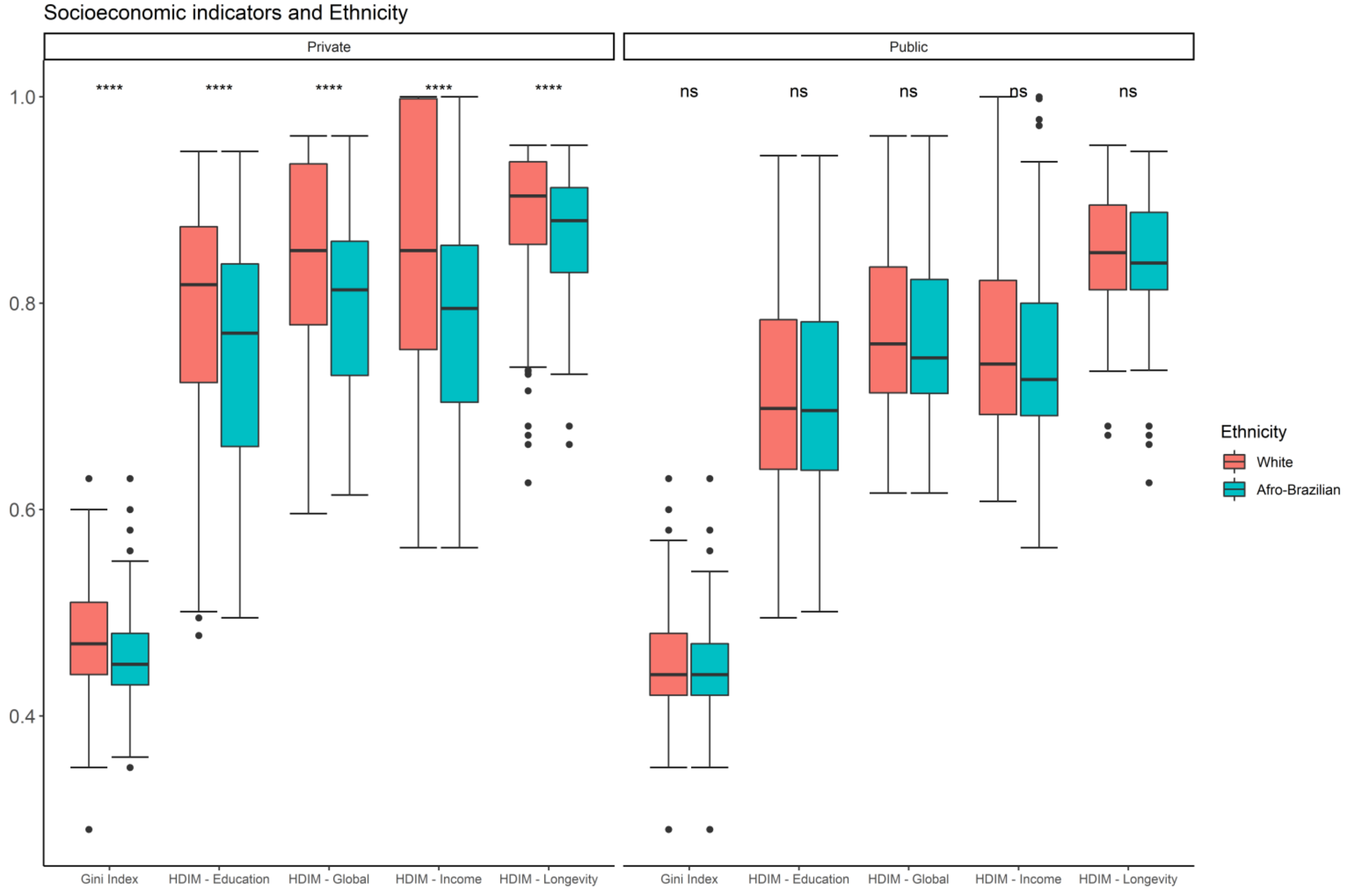

Figure S2. Distribution of socioeconomic data among different ethnic groups stratified by hospital administration. ***p < 0.001

6. Sensitive analysis – a) Large hospitals of private and public governance; b) adding an interaction term in the original model; c) adding hospitals number of beds in the original model

Considering that many patients were admitted to small hospitals and medical facilities of private administration, and there was no hospital with less than 50 beds in public administration, we performed an additional analysis considering only large hospitals with more than 151 beds. As a result, there were twenty large hospitals of private administration vs. twenty-one hospitals of public administration. Thus, the total number of patients in this subanalysis comprises 6993 patients, 6036 patients in private vs. 957 patients in public hospitals. eTable 3 depicts the characteristics of this cohort, and eTable 4 depicts the results of a shared frailty gamma Cox regression on survival in this cohort. Again, no significant differences were observed in this subgroup analysis compared with the entire cohort.

Additionally, we included an interaction term of socioeconomic factors, type of hospital, outcomes, and additional stratification of hospitals according to the number of beds. Including these interaction terms and factors did not significantly improve the model performance, as evidenced by the AIC values and the likelihood ratio test.

6.1 Table S4. Comparison of large hospitals of private vs. public governance

	Overall (n = 41)	Private (n = 20)	Public (n = 21)	p
n	n = 6993	n = 6036	n = 957	
Demographics				
Age (median [IQR])	72.00 [57.00–82.00]	75.00 [61.00–83.00]	52.00 [32.00–66.00]	<0.001
Male (%)	3870 (55.3)	3338 (55.3)	532 (55.6)	0.895
Nonwhite ethnicity (%)	1090 (15.6)	719 (11.9)	371 (38.8)	<0.001
AKI phenotype				
Acute-on-chronic kidney disease (%)	2221 (31.8)	2001 (33.2)	220 (23.0)	<0.001
Community-acquired AKI (%)	1968 (28.1)	1521 (25.2)	447 (46.7)	<0.001
Medical admission (%)	4783 (68.4)	4231 (70.1)	552 (57.7)	<0.001
ICU admission (%)	5562 (79.5)	4996 (82.8)	566 (59.1)	<0.001
Comorbidities				
Chronic heart diseases (%)	2207 (31.6)	2070 (34.3)	137 (14.3)	<0.001
Diabetes (%)	1294 (18.5)	1181 (19.6)	113 (11.8)	<0.001
Obstructive vascular disease (%)	818 (11.7)	751 (12.4)	67 (7.0)	<0.001
Chronic hepatic disease (%)	293 (4.2)	269 (4.5)	24 (2.5)	0.007
Hypertension (%)	2765 (39.5)	2513 (41.6)	252 (26.3)	<0.001
Neoplasia (%)	1246 (17.8)	1039 (17.2)	207 (21.6)	0.001
Charlson score (mean (SD))	2.01 (0.87)	2.06 (0.88)	1.72 (0.75)	<0.001
Precipitating causes of AKI				
Sepsis at admission (%)	2812 (40.2)	2378 (39.4)	434 (45.4)	0.001
Later sepsis (%)	1908 (27.3)	1788 (29.6)	120 (12.5)	<0.001
CRS type I (%)	984 (14.1)	902 (14.9)	82 (8.6)	<0.001
Hypovolemia (%)	2160 (30.9)	1870 (31.0)	290 (30.3)	0.701
Nephrotoxicity (%)	756 (10.8)	653 (10.8)	103 (10.8)	1.000

Urological conditions (%)	445 (6.4)	273 (4.5)	172 (18.0)	<0.001
Major surgery (%)	1143 (16.3)	1033 (17.1)	110 (11.5)	<0.001
CRS type II (%)	291 (4.2)	278 (4.6)	13 (1.4)	<0.001
Hepatorenal syndrome (%)	145 (2.1)	138 (2.3)	7 (0.7)	0.003
Number of causes (mean (SD))	1.50 (0.65)	1.51 (0.65)	1.42 (0.60)	<0.001
Indications for commencing RRT				
Oliguria (%)	5225 (74.7)	4587 (76.0)	638 (66.7)	<0.001
Acidosis (%)	5109 (73.1)	4488 (74.4)	621 (64.9)	<0.001
Azotemia (%)	5847 (83.6)	5053 (83.7)	794 (83.0)	0.594
Hypervolemia (%)	2630 (37.6)	2266 (37.5)	364 (38.0)	0.797
Hyperkalemia (%)	1534 (21.9)	1194 (19.8)	340 (35.5)	<0.001
Number of indications (mean (SD))	2.93 (1.02)	2.93 (0.99)	2.92 (1.20)	0.935
Organ failures				
Mechanical ventilation (%)	5129 (73.3)	4589 (76.0)	540 (56.4)	<0.001
Vasopressors (%)	4891 (69.9)	4416 (73.2)	475 (49.6)	<0.001
Additional failing organs (mean (SD))	2.03 (1.27)	2.09 (1.25)	1.63 (1.32)	<0.001
RRT modality (%)				<0.001
SLED	3094 (44.3)	2909 (48.3)	185 (19.4)	
IHD	2389 (34.2)	1909 (31.7)	480 (50.3)	
SCHD	1398 (20.0)	1147 (19.0)	251 (26.3)	
PD	103 (1.5)	64 (1.1)	39 (4.1)	
Socioeconomic indicators				
Gini (mean (SD))	0.47 (0.05)	0.48 (0.05)	0.45 (0.05)	<0.001
HDIM - Global (mean (SD))	0.84 (0.09)	0.85 (0.08)	0.77 (0.08)	<0.001
HDIM - Income (mean (SD))	0.85 (0.12)	0.86 (0.11)	0.76 (0.09)	<0.001
HDIM - Longevity (mean (SD))	0.89 (0.05)	0.90 (0.05)	0.85 (0.05)	<0.001
HDIM - Education (mean (SD))	0.79 (0.10)	0.80 (0.10)	0.71 (0.09)	<0.001
Income (mean (SD))	2224.57 (1717.40)	2395.71 (1743.41)	1145.15 (1016.36)	<0.001
Outcome (%)				<0.001
Mortality	4804 (68.7)	4235 (70.2)	569 (59.5)	
Discharged on chronic dialysis	484 (6.9)	403 (6.7)	81 (8.5)	
Unknown	295 (4.2)	241 (4.0)	54 (5.6)	
Partial recovery	831 (11.9)	640 (10.6)	191 (20.0)	
Total recovery	579 (8.3)	517 (8.6)	62 (6.5)	

AKI: acute kidney injury; ICU: Intensive care unit; CRS: cardiorenal syndrome; RRT: renal replacement therapy; SLED: sustained low-efficiency dialysis; HD: intermittent hemodialysis; SCHD: slow-continuous hemodialysis; PD: peritoneal dialysis, HDMI: human development index (municipality); SD: standard deviation.

6.2 Table S5. Survival according to a frailty gamma Cox regression in the large hospitals' cohort

		all	HR (univariable), p	HR (multivariable), p
Age	Mean (SD)	67.4 (20.1)	1.01 (1.01–1.01, p < 0.001)	1.01 (1.01–1.01, p < 0.001)
Gender	Female	3123 (100.0)	-	-
	Male	3870 (100.0)	0.90 (0.85–0.95, p < 0.001)	0.93 (0.87–0.98, p = 0.008)
Ethnicity	White	5904 (100.0)	-	-
	Nonwhite	1089 (100.0)	0.92 (0.85–1.00, p = 0.055)	1.03 (0.95–1.13, p = 0.449)
Hospital governance	Private	6036 (100.0)	-	-
	Public	957 (100.0)	0.94 (0.86–1.03, p = 0.188)	1.05 (0.82–1.35, p = 0.698)
AKI phenotype I	Acute	4772 (100.0)	-	-
	Acute-on-chronic kidney disease	2221 (100.0)	0.74 (0.69–0.79, p < 0.001)	0.84 (0.79–0.90, p < 0.001)
AKI phenotype II	Hospital-acquired	5025 (100.0)	-	-
	Community-acquired	1968 (100.0)	0.66 (0.62–0.71, p < 0.001)	0.86 (0.80–0.93, p < 0.001)
AKI phenotype III	Nonseptic AKI	4181 (100.0)	-	-
	Septic AKI	2812 (100.0)	1.31 (1.24–1.39, p < 0.001)	1.16 (1.09–1.23, p < 0.001)
AKI phenotype IV	Nonoliguric AKI	1768 (100.0)	-	-
	Oliguric AKI	5225 (100.0)	1.75 (1.63–1.88, p < 0.001)	1.43 (1.33–1.54, p < 0.001)
Number of organ failures (per-organ)	Mean (SD)	2.0 (1.3)	1.39 (1.35–1.42, p < 0.001)	1.35 (1.32–1.39, p < 0.001)
Charlson score (per-point)	Mean (SD)	2.0 (0.9)	1.04 (1.01–1.08, p = 0.009)	1.03 (1.00–1.07, p = 0.078)
Human development Index	Low	1600 (100.0)	-	-
	High	5393 (100.0)	1.10 (1.03–1.18, p = 0.007)	0.99 (0.89–1.09, p = 0.798)
Income	Low	2334 (100.0)	-	-
	High	4659 (100.0)	1.10 (1.04–1.17, p = 0.001)	1.02 (0.92–1.12, p = 0.707)
Gini index	(0.29, 0.45)	2933 (100.0)	-	-
	(0.45, 0.49)	1875 (100.0)	1.03 (0.96–1.11, p = 0.393)	1.02 (0.95–1.10, p = 0.580)
	(0.49, 0.63)	2185 (100.0)	1.11 (1.04–1.19, p = 0.002)	0.98 (0.91–1.06, p = 0.597)
Frailty (hospital)			-	-

AKI: acute kidney injury; SD: standard deviation,

6.3 Table S6. Addition of an interaction term (socioeconomic factors*hospital type*outcomes) and number of beds in the model

		HR (multivariable), p
Age	Mean (SD)	1.007 (1.0057–1.0082, p < 0.001)
Gender	Female	-
	Male	0.92 (0.89–0.96, p <0.001)
Ethnicity	White	-
	Nonwhite	0.98 (0.93–1.04, p = 0.709)
Hospital governance	Private	-
	Public	1.01 (0.82–1.23, p = 0.913)

AKI phenotype I	Acute	-
	Acute-on-chronic kidney disease	0.78 (0.75–0.81, p < 0.001)
AKI phenotype II	Hospital-acquired	-
	Community-acquired	0.68 (0.65–0.72, p < 0.001)
AKI phenotype III	Nonseptic AKI	-
	Septic AKI	1.22 (1.17–1.27, p < 0.001)
AKI phenotype IV	Nonoliguric AKI	-
	Oliguric AKI	1.48 (1.41–1.56, p < 0.001)
Number of organ failures (per-organ)	Mean (SD)	1.04 (1.01–1.07, p < 0.001)
Charlson score (per-point)	Mean (SD)	0.99 (0.97–1.01, p = 0.635)
Human development Index	Low	-
	High	0.95 (0.80–1.12, p = 0.554)
Income	Low	-
	High	1.05 (0.99–1.12, p = 0.078)
Gini index	(0.29, 0.45)	-
	(0.45, 0.49)	1.01 (0.95–1.05, p = 0.784)
	(0.49, 0.63)	0.99 (0.94–1.04, p = 0.766)
Hospital Size	High	-
	Medium	1.06 (0.89 – 1.28, p=0.466)
	Small	0.98 (0.80 – 1.19, p=0.858)
Socioeconomic fators * Hospital Types * Outcomes	Interaction term	1.05 (0.88 – 1.25, p=0.547)

AKI: acute kidney injury; SD: standard deviation,

Likelihood ratio test:

> anova(model, model1) → Analysis of Deviance p=0.6366

7. Table S7. Discharge outcomes among survivors, stratified by hospital administration and socioeconomic indicators.

a) Stratified by hospital administration							
	All (n = 4415)	Private (n = 3995)	Public (n = 420)	p value			
Dialysis dependence	1034 (23.4)	945 (23.7)	89 (21.2)	<0.001			
Partial recovery	1416 (32.1)	1210 (30.3)	206 (49.0)				
Complete recovery	1179 (26.7)	1112 (27.8)	67 (16.0)				
Unknown	786 (17.8)	728 (18.2)	58 (13.8)				
b) Stratified by socioeconomic indicators							
		Overall	Dialysis dependence	Partial recovery	Complete recovery	Unknown	p value

n (%)		4415 (100)	1034 (23.4)	1416 (32.1)	1179 (26.7)	786 (17.8)	
HDIM (mean (SD))		0.82 (0.09)	0.82 (0.09)	0.82 (0.09)	0.83 (0.09)	0.83 (0.09)	0.053
Income (%)	Low	1841 (41.7)	450 (43.5)	602 (42.5)	459 (38.9)	330 (42.0)	0.136
	High	2574 (58.3)	584 (56.5)	814 (57.5)	720 (61.1)	456 (58.0)	
Gini Index (%)	(0.29, 0.44)	1657 (37.5)	416 (40.2)	519 (36.7)	428 (36.3)	294 (37.4)	0.362
	(0.44, 0.48)	1415 (32.0)	329 (31.8)	448 (31.6)	379 (32.1)	259 (33.0)	
	(0.48, 0.63)	1343 (30.4)	289 (27.9)	449 (31.7)	372 (31.6)	233 (29.6)	

HDIM: human development index (municipality); SD: standard deviation.

APPENDEX

STROBE Statement—checklist of items that should be included in reports of observational studies

	Item No	Recommendation	Page No
**Title and abstract**	1	(*a*) Indicate the study's design with a commonly used term in the title or the abstract	1
		(*b*) Provide in the abstract an informative and balanced summary of what was done and what was found	2
**Introduction**			
Background/rationale	2	Explain the scientific background and rationale for the investigation being reported	3
Objectives	3	State specific objectives, including any prespecified hypotheses	3
**Methods**			
Study design	4	Present key elements of study design early in the paper	4
Setting	5	Describe the setting, locations, and relevant dates, including periods of recruitment, exposure, follow-up, and data collection	4
Participants	6	(*a*) *Cohort study*—Give the eligibility criteria, and the sources and methods of selection of participants. Describe methods of follow-up<br>*Case-control study*—Give the eligibility criteria, and the sources and methods of case ascertainment and control selection. Give the rationale for the choice of cases and controls<br>*Cross-sectional study*—Give the eligibility criteria, and the sources and methods of selection of participants	4

		(*b*) *Cohort study*—For matched studies, give matching criteria and number of exposed and unexposed<br>*Case-control study*—For matched studies, give matching criteria and the number of controls per case	4
Variables	7	Clearly define all outcomes, exposures, predictors, potential confounders, and effect modifiers. Give diagnostic criteria, if applicable	4,5
Data sources/ measurement	8*	For each variable of interest, give sources of data and details of methods of assessment (measurement). Describe comparability of assessment methods if there is more than one group	*4,5*
Bias	9	Describe any efforts to address potential sources of bias	11,12
Study size	10	Explain how the study size was arrived at	4
Quantitative variables	11	Explain how quantitative variables were handled in the analyses. If applicable, describe which groupings were chosen and why	4,5
Statistical methods	12	(*a*) Describe all statistical methods, including those used to control for confounding	6
		(*b*) Describe any methods used to examine subgroups and interactions	6
		(*c*) Explain how missing data were addressed	6
		(*d*) *Cohort study*—If applicable, explain how loss to follow-up was addressed<br>*Case-control study*—If applicable, explain how matching of cases and controls was addressed<br>*Cross-sectional study*—If applicable, describe analytical methods taking account of sampling strategy	6
		(*e*) Describe any sensitivity analyses	6

Results

Participants	13*	(a) Report numbers of individuals at each stage of study—eg numbers potentially eligible, examined for eligibility, confirmed eligible, included in the study, completing follow-up, and analysed	4, 7
		(b) Give reasons for non-participation at each stage	N/A
		(c) Consider use of a flow diagram	4
Descriptive data	14*	(a) Give characteristics of study participants (eg demographic, clinical, social) and information on exposures and potential confounders	7
		(b) Indicate number of participants with missing data for each variable of interest	4

		(c) *Cohort study*—Summarise follow-up time (eg, average and total amount)	4,7
Outcome data	15*	*Cohort study*—Report numbers of outcome events or summary measures over time	*8,9*
		Case-control study—Report numbers in each exposure category, or summary measures of exposure	
		Cross-sectional study—Report numbers of outcome events or summary measures	
Main results	16	(*a*) Give unadjusted estimates and, if applicable, confounder-adjusted estimates and their precision (eg, 95% confidence interval). Make clear which confounders were adjusted for and why they were included	8,9
		(*b*) Report category boundaries when continuous variables were categorized	5
		(*c*) If relevant, consider translating estimates of relative risk into absolute risk for a meaningful time period	N/A
Other analyses	17	Report other analyses done—eg analyses of subgroups and interactions, and sensitivity analyses	7
**Discussion**			
Key results	18	Summarise key results with reference to study objectives	10
Limitations	19	Discuss limitations of the study, taking into account sources of potential bias or imprecision. Discuss both direction and magnitude of any potential bias	11
Interpretation	20	Give a cautious overall interpretation of results considering objectives, limitations, multiplicity of analyses, results from similar studies, and other relevant evidence	10,11
Generalisability	21	Discuss the generalisability (external validity) of the study results	12
**Other information**			
Funding	22	Give the source of funding and the role of the funders for the present study and, if applicable, for the original study on which the present article is based	13
